# Supplementary material for: A simple, scalable approach to building a cross-platform transcriptome atlas
Source: PLoS Comput Biol. 2020 Sep 28;16(9):e1008219. doi: 10.1371/journal.pcbi.1008219 (PMC7544119; doi:10.1371/journal.pcbi.1008219)
Supplement: S1 Text — Expanded description of the gene filtering, rank transformation and stability analysis. (PDF) [file pcbi.1008219.s001.pdf]

S1 SUPPLEMENTARY METHOD

S1.1 Platform Effect Analysis and Gene Selection

S1.1.1 Choosing the platform-variance threshold

The appropriate value of the threshold is set by assessing the platform-batch effect after progressively lowering the threshold. A PCA using a threshold of 0.2 is shown in Figure 3 **D**. Compared to Figure 3 A the effect of platform is effectively suppressed and samples now cluster according to the annotated biological type. As the threshold is lowered, fewer genes pass the cut and are used to generate the PCA graphs. However, once platform is removed there is a range of thresholds for which the biological structure remains stable.

The effect of lowering the threshold is shown in Fig 3 and Fig S3. Figure 3 depicts the changing PCA as the threshold is lowered from 0.8 down to 0.2. The platforms appear separated in **A**, but merge by **D**. Similar observations can be obtained from Fig S3. As we lower the threshold (y-axis), we measure the platform effect for each of the first 10 PCA components independently using the Kruskal-Wallis H (KWH) Test (as implemented in Jones et al., 2001). We consider the first 10 principal components in this analysis, as they extend well past the ’elbow’ of the respective scree plot of the PCA, located at approximately 3-4 principal components. The choice covers the most relevant principal components and shows their relation to the platform effect as the threshold is lowered. Note that we do not retain all 10 components in our final PCA results, only those which demonstrate a reduced platform effect.

The KHW test quantifies the difference in median between the different populations. Darker boxes indicate less platform dependence, and vice-versa. Lowering the threshold has the effect of a) firstly moving the main platform dependence from component 1 to lower components, and b) eventually suppressing the effect over all components. This agrees with the visual inspection of the PCA after filtering genes with a range of thresholds. KWH Test values of $\sim$0.2 are present on the first 3 principal components when the threshold reaches ≤ 0.2. Empirically, it is a good indicator that the platform effect is absent and the first 3 principal components form the PCA.

The platforms we use are Affymetrix HuGene and U133 Plus 2, RNA sequencing of any version, Illumina V4, Illumina V2 microarrays. These are the platforms with blood related datasets spanning several subtypes. Note that different versions of a platform, e.g. HuGene version 1 and 2, tend to cluster together as if they were one. The difference between them is small compared to the effect between the other platform types, and is also small compared to the effect of the biology. These differing versions are placed in the same category and treated as one platform. That leads to five platforms categories listed above.

S1.2 Stability and H-Index

Assessment of the stability of the gene selection using two random sampling techniques - bootstrap resampling directly upon the samples and leave-one-out resampling applied to datasets. Bootstrap resampling was performed 500 times. Multiple clustering methods were applied, in order to avoid results being dependent upon an idiosyncrasy of one algorithm. Our goal is not to assess clustering algorithms, but rather provide evidence that the clustering structure present in the PCA is stable. Clustering is performing by KMeans and Hierarchical clustering implemented in cite Sklearn.

The H-Index (as described in (Shannon et al., 2016)) was calculated for each cluster, with the base case being the data with no resampling. At each iteration the process is repeated starting from the calculation of platform dependence: the univariate linear model calculated, thresholded with a value of 0.2 to select a subset of genes, PCA generated, and clustering algorithm applied. Tables S3 and S4 show the results for a selection of different clustering algorithms, and number of clusters. Results in the tables below show, for each combination of clustering algorithm and number of classes, the median H-index across all clusters, and the maximum and minimum of the H-index across all clusters.

We also can consider the stability of genes included under resampling. For each resample iteration, we calculated the percentage of genes that are still pass the cut.

Datasets are generated under different conditions and platforms, and in this way can be considered a single data point. Thus, resampling can be performed on either individual samples or datasets. This is further complicated as some datasets contribute a large number of samples. We list the results of leave-one-out resampling on datasets, followed by bootstrapping the samples, as bootstrapping datasets can have an extremely large effect on the underlying composition of the data.

Non-Blood Samples

Non-blood related samples were taken from publicly available data in Stemformatics. It was obtained by searching for sample types containing any one of the following terms: iPSC, embryonic, mesenchymal, mesoderm, fibroblast, pluripotent, neuron, astrocyte, adipocyte, melanocyte, epithelium, neural, endoderm, cardiomyocyte. The are a couple of iPSC derived blood datasets, which were excluded. The results of this search was 2093 samples from 140 datasets. The Stemformatics dataset id, the title of the relevant publication and the number of samples drawn from each dataset is listed in the table non_blood_datasets.tsv.

S1.3 Rank transformation corrects for platform variation between studies

The percentile rank transformation adopted in this study is advantageous as a non-parametric technique that does not require any specification of the potential batch sources or the experimental study design. This transformation is thus appropriate for any unknown confounding factor that was not reported in a

given study, and can be considered as a normalisation technique in our atlas to enable between-study comparisons.

We simulated studies with 1000 samples, 10000 genes for 10 cell types and 4 batches. Amongst the 10000 genes, 100 to 200 genes were simulated as differentially expressed with a cell type effect. Count data were generated using negative binominal distributions with genewise specified dispersion trend described by Law et al. (2014). Batch effects were then included in all genes by applying 4 different non-linear monotonic functions to the simulated data. Figure S5 shows that the percentile rank transformation outperforms two popular batch effect correction methods, limma and Combat (Ritchie et al., 2015; Johnson et al., 2006).
