# Supplementary figures and images for: A simple, scalable approach to building a cross-platform transcriptome atlas
Source: PLoS Comput Biol. 2020 Sep 28;16(9):e1008219. doi: 10.1371/journal.pcbi.1008219 (PMC7544119; doi:10.1371/journal.pcbi.1008219)

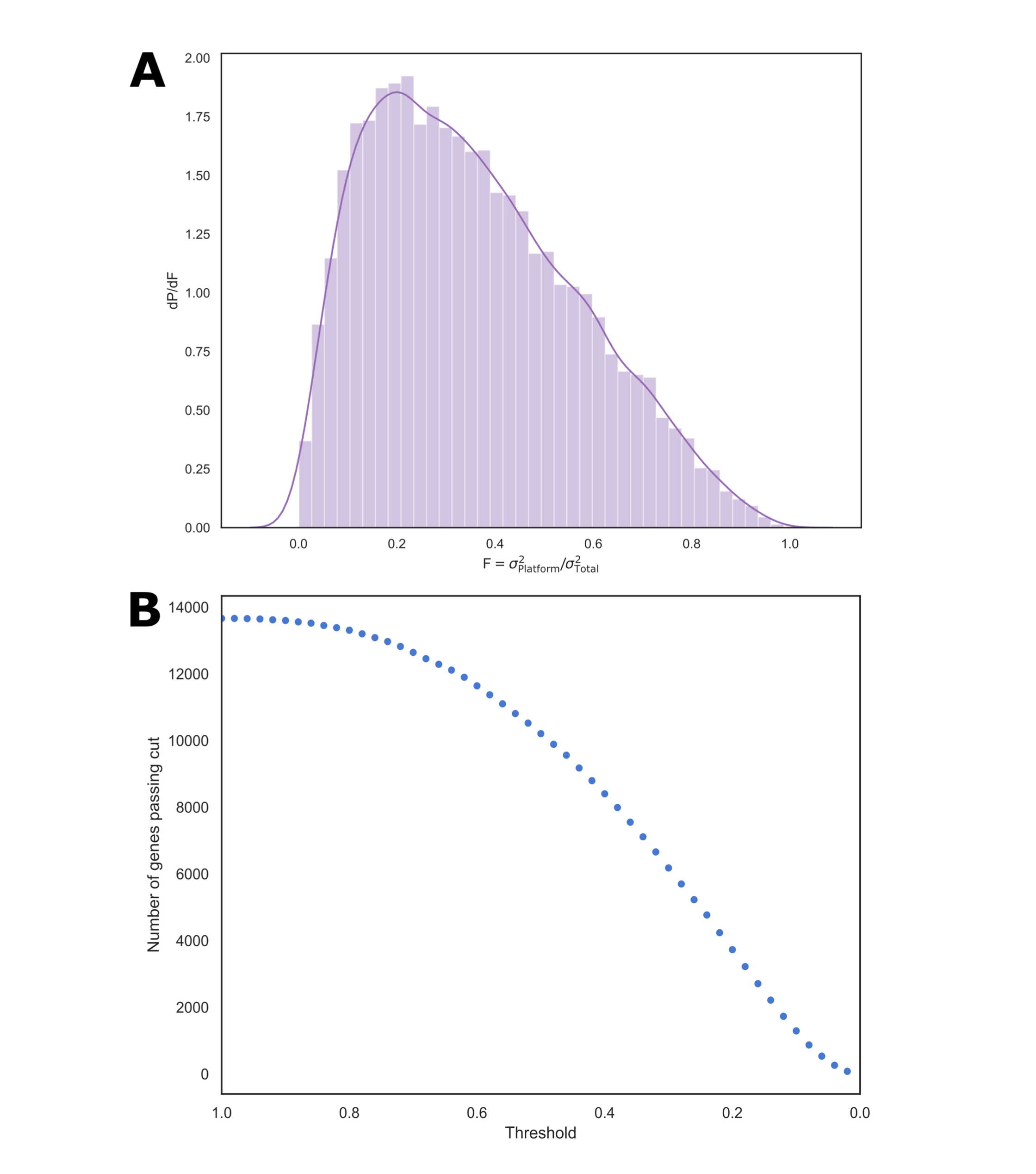

Supplement: S1 Fig — A: The distribution of the fraction of variance attributable to platform for the blood data. It is weighted towards low ratios, indicating that biological variation forms a major part of the signal. B:The total number of genes that pass the cut for platform variance thresholds from 1.0 to 0. (TIFF) [file pcbi.1008219.s002.tiff]

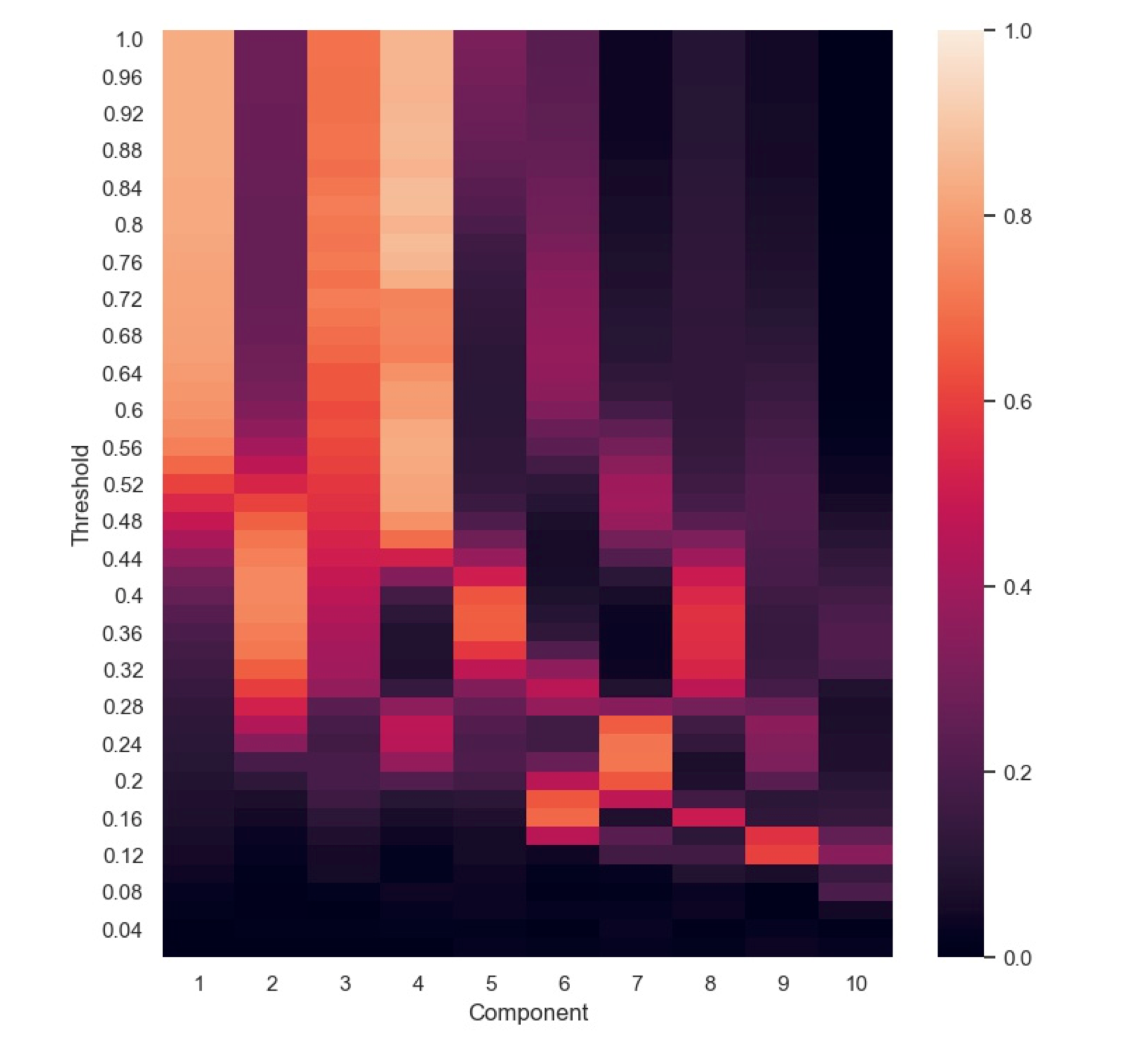

Supplement: S2 Fig — Lower values for the KWH test indicate platform has less effect upon the component. Lowering the threshold has the effect of both moving platform-related components to lower components, and decreasing the overall dependence upon platform. (TIFF) [file pcbi.1008219.s003.tiff]

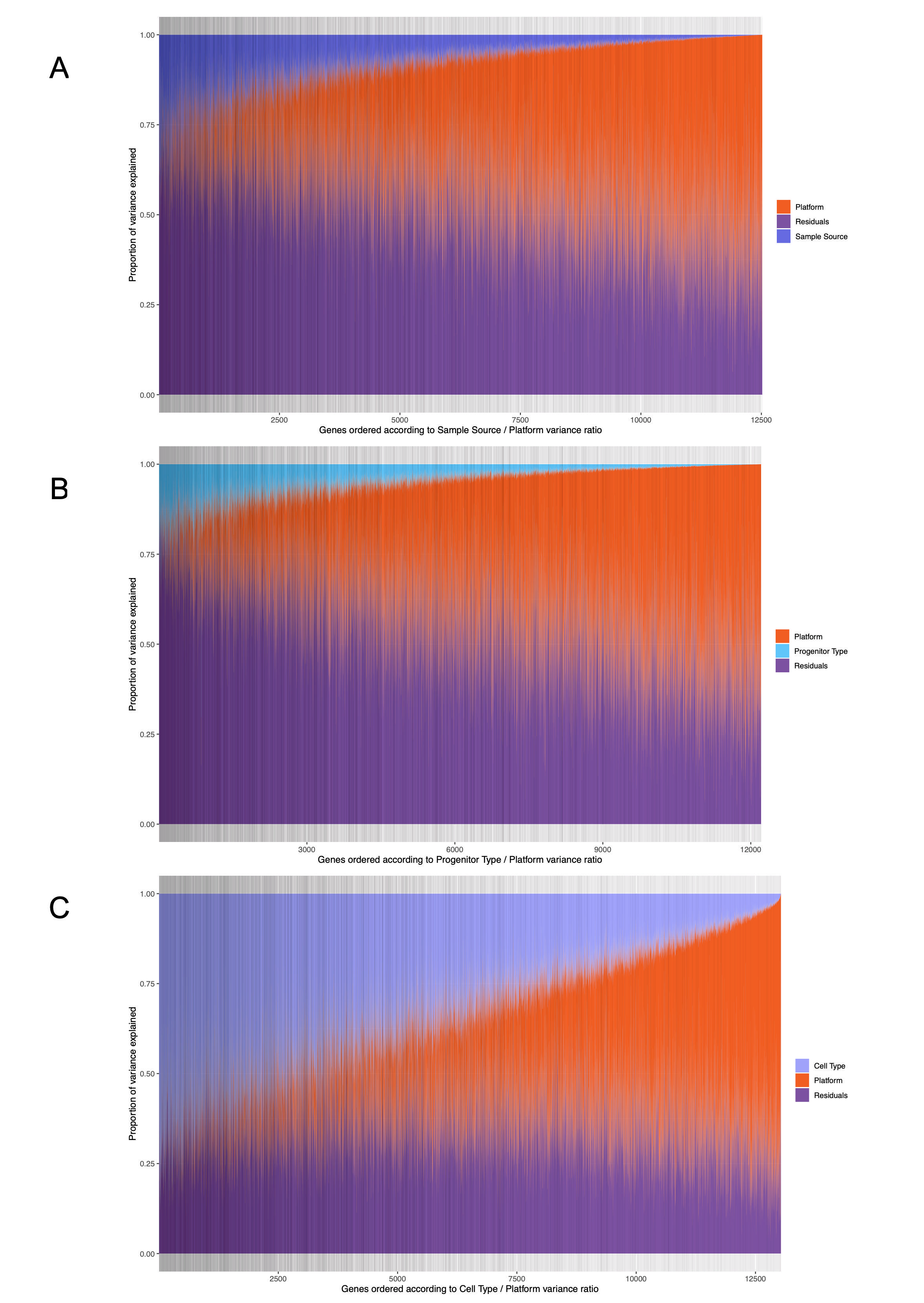

Supplement: S3 Fig — Each gene is depicted as a vertical line on the x-axis, and genes are ranked according to the ratio Sample Source / Platform explained variance. Dark gray vertical lines indicate genes that were retained in the filtered data set. (TIFF) [file pcbi.1008219.s004.tiff]

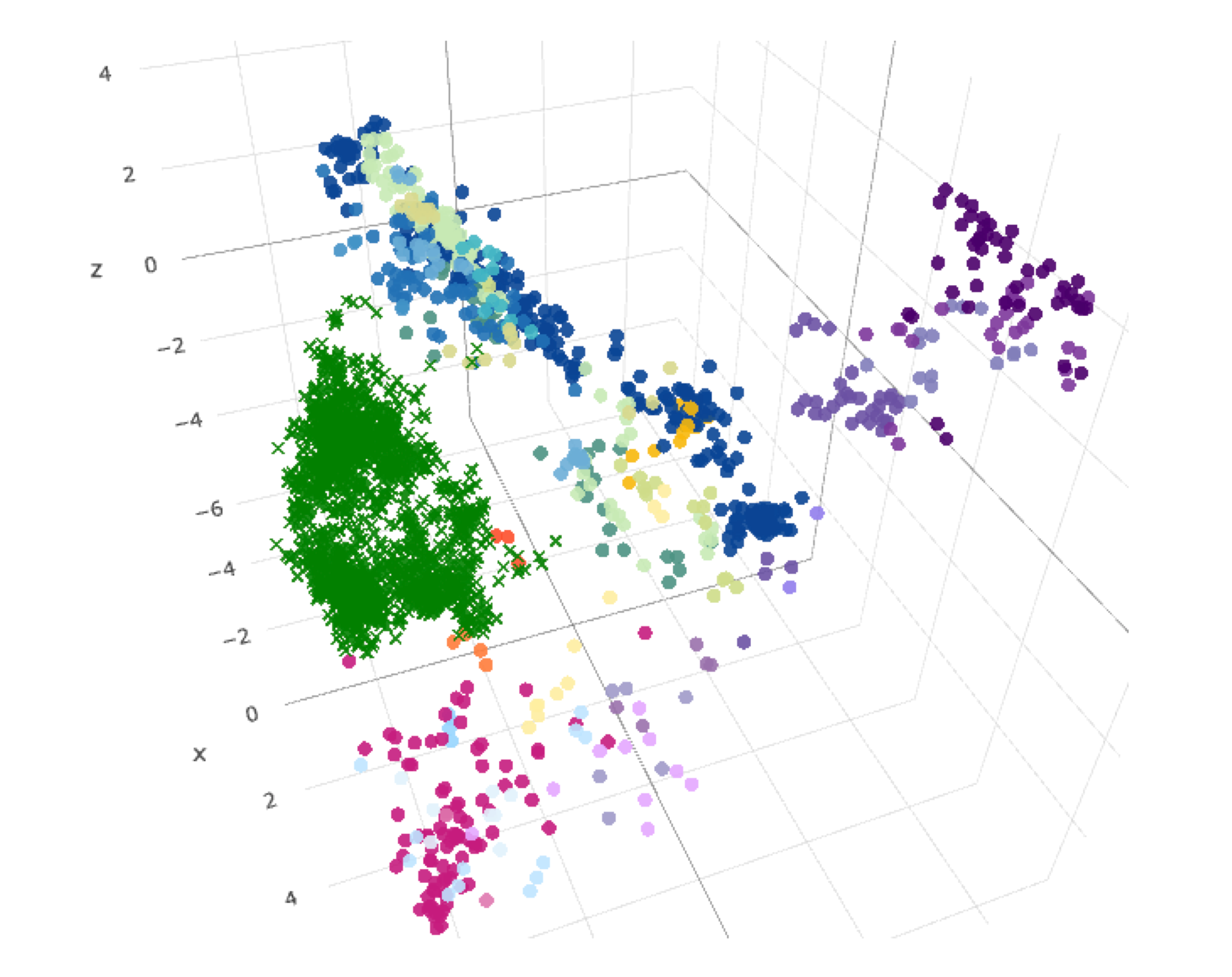

Supplement: S4 Fig — They are displayed as green crosses. They sit in a region low on component 2, a region not populated by either by the blood samples used to generate the atlas. (TIFF) [file pcbi.1008219.s005.tiff]

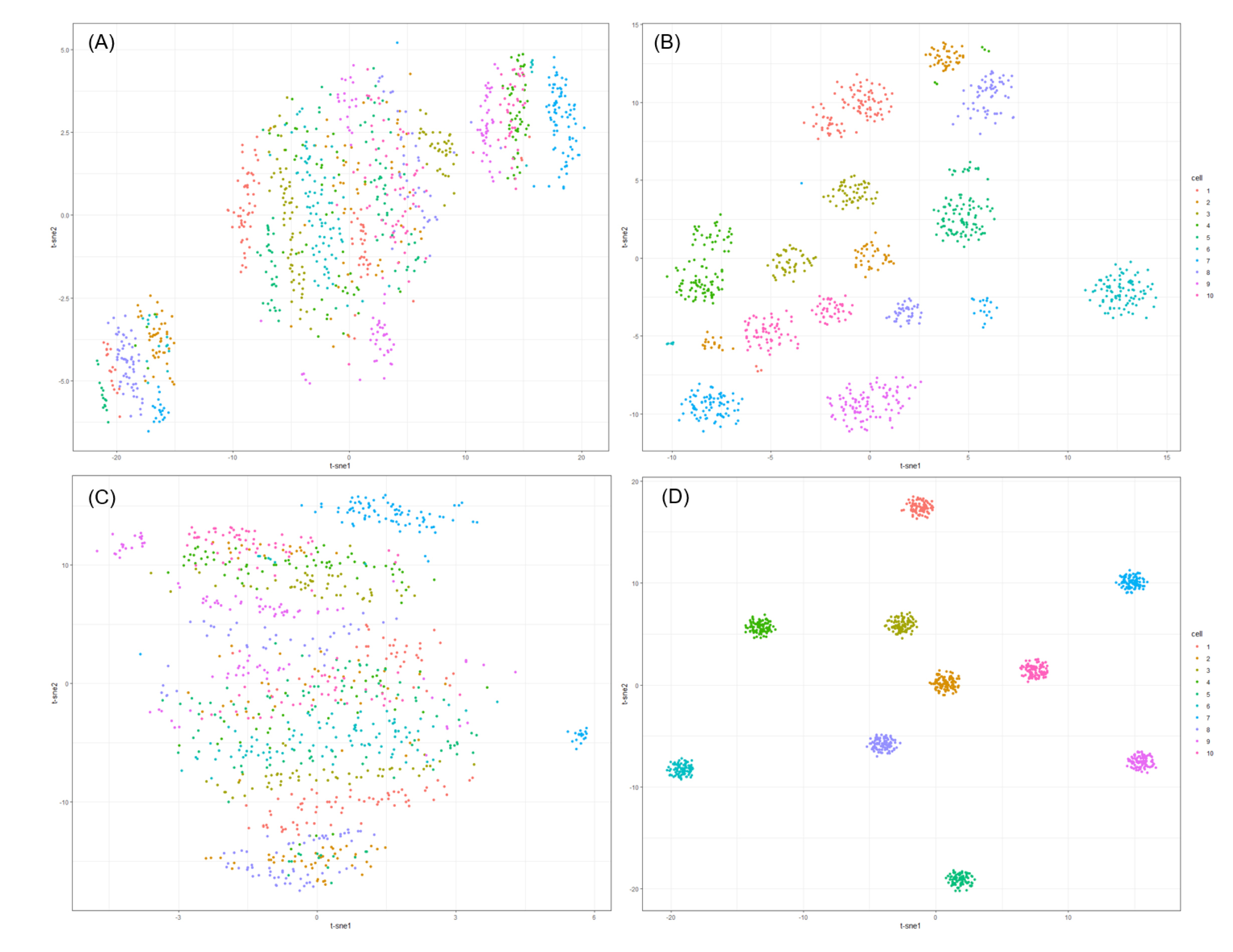

Supplement: S5 Fig — Ten cell types are indicated by colors. (A): original count data include a batch effect across 4 platforms. (B): correction for platform effect with limma followed by voom transformation, (C): Combat and (D): percentile rank transformation. (TIFF) [file pcbi.1008219.s006.tiff]
