# Supplementary material for: A simple, scalable approach to building a cross-platform transcriptome atlas
Source: PLoS Comput Biol. 2020 Sep 28;16(9):e1008219. doi: 10.1371/journal.pcbi.1008219 (PMC7544119; doi:10.1371/journal.pcbi.1008219)
Supplement: S2 Table — (DOCX) [file pcbi.1008219.s008.docx]

| Cluster  Number | Cluster Name | Total  Samples | Cell Type Identity |
| --- | --- | --- | --- |
| 1 | Lymphocyte cluster | 120 | B Cell (30/30), natural killer cell (15/15), T Cell  (72/72), natural killer progenitor (3/4) |
| 2 | Circulating Mono-  cyte and Granulocyte | 126 | monocyte (111/284), granulocyte (10/10), neu-  trophil (4/4), macrophage (1/104) |
| 3 | Progenitor | 146 | MK (4/4), erythrocyte (4/7), **HPC** (91/92), **CMP**  (12/12) GMP (13/14), LP (22/25) |
| 4 | Macrophage | 275 | monocyte (173/284), macrophage (92/104), den-  dritic cell (10/172) |
| 5 | Dendritic Cell | 106 | dendritic cell (105/172), macrophage (1/104) |
| 6 | Mixed | 77 | dendritic cell (57/172), microglia (10/25), progeni-  tor (10/96) |
