# Supplementary material for: A simple, scalable approach to building a cross-platform transcriptome atlas
Source: PLoS Comput Biol. 2020 Sep 28;16(9):e1008219. doi: 10.1371/journal.pcbi.1008219 (PMC7544119; doi:10.1371/journal.pcbi.1008219)
Supplement: Attachment — Submitted filename: Angel_response to reviewers.pdf [file pcbi.1008219.s011.pdf]

We appreciate the careful and constructive critique by both reviewers, and our responses are appended here. I also enclose a fully marked up version indicating where changes to the text have been included.

The major changes to the manuscript are

1. To place more emphasis on the resource we have now extended the discussion with suggested use cases of the atlas.

2. We have added an additional figure (Fig 3) in line with reviewer 2's recommendations of moving the batch correction modelling from the supplement to the main manuscript text.

3. We provide a vignette for suggested handling of single cell data and projection to the atlas.

Reviewer #1:
It is an excellent integrative analysis tool which should enable the re-use of a significant quantity of the transcriptomic data that has already been generated via older methods such as micro-arrays and bulk RNAseq and also provides possibilities for integrative analysis in the scRNAseq space
Your paper explains your method clearly explaining both its advantages compared to other approaches and its drawbacks.
You have made the methods and the data all openly available via the https://www.stemformatics.org/ and code sharing repos

We thank Dr Clark for her careful reading of the manuscript and suggested improvements to readability. We have adopted all of the following suggestions.

Page 3/24 - Line 9 "Yet technological change drives much recursive data derivation"

page 3/24 - Line 12 "catalogue"

page 5/24 - Line 23 "Gene expression for each sample"

page 5/24 - Line 24 "the gene with the highest expression is assigned a value of 1 and the lowest receives a value of 0"

page 18/24 - Line 25
"by only a few data sets" The stray 2 was meant to specify Figure 2 (with apologies for the LaTex formatting issue) and has been amended to refer to the Figure.

Thank you for providing this excellent analysis and software to the community. I look forward to seeing it more broadly used to support single cell analysis.

We are grateful for the strong endorsement of our approach.

Reviewer #2: *Angel et al. present a resource that combines a large number of hematopoietic cell profiling datasets through a simple and effective normalization method, offered through an online portal and open*

Professor Christine Wells
Director
Centre for Stem Cell Systems
School of Biomedical Sciences, Faculty of Medicine, Dentistry and Health
30 Royal Parade, The University of Melbourne, Victoria 3010 Australia
T: +61 3 83443795 | M: +61 478306783 | E: wells.c@unimelb.edu.au | W: Stemformatics.org | **unimelb.edu.au**

source code. This could be a valuable resource, and while is currently limited in scope to hematopoiesis, is a great proof of principle of combining disparate datasets for biological analysis. I have several questions or concerns about the implementation of this atlas that are listed below.

1. Platform effect.
I appreciate the method the authors use to deconvolve platform effect - it is intuitive, and it also seems sensible to use subsets of genes, since using very large gene sets (tens of thousands) for analyses tends to decrease the signal-to-noise. However in the main manuscript there is little emphasis on the platform effect and the impact of the choice of gene set. It would also be helpful to have more detail about the impact of the size of the input gene set on the PCA (as in S4 or similar) - what variance threshold corresponds to 500 or 5000 genes, etc?

We agree with the reviewer's suggestions, and in response to this request have moved the platform supplementary data into the main manuscript. To do so, we have created an additional figure (Figure 3, P11) which elaborates on what was Figure S4. The number of genes impacted by the variance filtering is now added as panel B in supplementary figure 1B.

We have also added the following text to P11 the results section, to address the impact of variance threshold on platform effect and on gene filtering.

"Figure 3 shows the process of implementing progressively stricter thresholds to generate the PCA. Beginning with a permissive threshold of 0.8 platform contribution to total variance in panel A, the separation of platforms in the PCA space is clearly evident. As the threshold is lowered to 0.6, 0.4 and 0.2 (panels B, C and D), samples from the different platforms mix and form new clusters. This in turn impacts on the number of genes available to construct the PCA, illustrated in Figure S1B. A threshold of 0.5 restricts subsequent analyses to approximately 10000 genes, a threshold of 0.25 approximately 5000 genes, and when the threshold is as strict as 0.05, only 500 genes remain."

*Also: if datasets between platforms differ by *biological* variation, e.g. different (proportions of) cell types, how does the variance threshold handle this?*

We address this in the original manuscript by assessing the stability of the sample clustering when iteratively dropping individual datasets out of the study, including large myeloid-specific or lymphocyte-specific studies. Please see the section on page 15, and Tables S3 and S4. We also provide the example of a much less biologically diverse sub-atlas (moving from the more diverse blood to less biologically diverse myeloid cells), noting that while different genes are represented in the myeloid atlas, the total number of genes approximate the larger atlas.

Class balance is an important consideration in supervised normalisation approaches such as COMBAT. The method described in the current manuscript can tolerate class imbalance in individual datasets but nonetheless does require consideration of sample type representation across platforms. As can be seen from the examples above, not every sample class requires representation in every individual experiment, nor every platform, but sufficient overlap of major classes is required for a stable representation of the biology.

We agree with the reviewer that this is an important point, and to make this clearer we've added the following section to page 11 to emphasis consideration of experimental design and the potential for this to impact on variance modelling.

Professor Christine Wells
Director
Centre for Stem Cell Systems
School of Biomedical Sciences, Faculty of Medicine, Dentistry and Health
30 Royal Parade, The University of Melbourne, Victoria 3010 Australia
T: +61 3 83443795 | M: +61 478306783 | E: wells.c@unimelb.edu.au | W: Stemformatics.org | **unimelb.edu.au**

"It is important to note that the distribution of samples on the PCA is a function of the biology of the samples, combined with the systematic effect of the platform upon genes. If that biology is not well represented, variance modelling will be more difficult. In order to uncover more fine grain detail, it is necessary to refine the set of samples for class and platform representation and recursively apply this process."

2. PCA. Why were 10 principal components chosen? Is there justification for their use or any comparison with other possible choices?
We confess that this was a somewhat arbitrary choice. We looked at the first 10 PCA as they extended well past the 'elbow' of major variance in the scree plots for successive atlas iterations. We've added this explanation to the Supplementary text:

"We consider the first 10 principal components in this analysis, as they extend well past the 'elbow' of the respective scree plot of the PCA, located at approximately 3-4 principal components. The choice covers the most relevant principal components and shows their relation to the platform effect as the threshold is lowered. Note that we do not retain all 10 components in our final PCA results, only those which demonstrate a reduced platform effect."

3. Details of biological datasets included are lacking, e.g. disease condition? I notice that some datasets describe pathologies (e.g. PMID 27630125, CML): would pathological myeloid cell populations not be expected to lie far from healthy hematopoietic populations? Is there a way to tag disease states and other features on the atlas?

We apologise for the poor description of our data curation in the original manuscript. We've added clarification to the methods section on sample curation, adding the line "Not all samples were used from each dataset to construct the atlas, which excluded cells from blood pathologies such as leukemic cell types" to P5.

We further added a paragraph into the discussion (P18) that reads:
"That this multi-scaled nature of a biological system can be captured recursively provides us with new opportunities to review phenotypes that might be expected to deviate from a reference atlas. Examples of future atlases might include disease states that fundamentally alter cell state, or experimental manipulation that creates new cell types. For these applications we recommend a multi-tiered approach, first projecting the new cell types to the current reference atlas to assess similarity to the groups included in the reference, then recompiling the atlas with the disease samples included to allow for additional biological variance specific to the disease to be captured in the new atlas. A leukemia atlas, for example, would comprise both healthy and leukemic cell types. An inflammatory atlas would include naive and activated cell types, and so on. The critical consideration here is adequate replication across data sets of the sample categories that are included in any rederived atlas."

4. In more general terms, while I do see the value of this resource, what are the use-cases that the authors imagine? E.g. biological discovery? In which case one would need additional labelling of the samples, by cell type or disease state.
We thank the reviewer for urging us to place more emphasis on the resource and it's use cases. The most common scenario is the rapid ability for users to review the behaviour of external samples against an independent reference, providing confidence in specificity of markers or phenotype. Our own group uses it to compare pluripotent stem cell derived models to their in vivo counterparts (and we refer the reviewer to

Professor Christine Wells
Director
Centre for Stem Cell Systems
School of Biomedical Sciences, Faculty of Medicine, Dentistry and Health
30 Royal Parade, The University of Melbourne, Victoria 3010 Australia
T: +61 3 83443795 | M: +61 478306783 | E: wells.c@unimelb.edu.au | W: Stemformatics.org | **unimelb.edu.au**

the accompanying preprint from Rajab et al). In addition to the new discussion paragraphs included in response to the reviewer above (point 3) we add the following paragraphs to the conclusion (P20) to elaborate on these use cases.

"This allows users to rapidly review gene expression across a large number of samples to find reproducible markers of cell type, and new markers correlated to derivation method, culture condition, or extrinsic signals.

Recursive application of the method was demonstrated by the general categorisation seen in whole blood to the identification of specific myeloid cell types and activation states in the accompanying myeloid atlas. The projection of additional data onto the atlas, provides a tool for researchers to compare their own data to a robust reference collection. Projection of single cell data provides definitive annotations of blood cell clusters without prior assignment of marker genes in the scRNA-seq data.

The method is simple and scalable, so providing anyone with the means to curate their own reference atlas to address additional biological systems. Implementation of the blood and myeloid atlases provides a simple web-based tool in the Stemformatics platform."

5. It is unclear how single-cell datasets are handled. Please expand on the "pseudo-bulk" aggregation is performed. Moreover, the projection of new single-cell datasets onto this atlas is likely to be a popular task, given the amount of interest in this area, so other examples would be extremely helpful.

What is minimum "sample" size (i.e. # of aggregated single cells)? Could single cells be rank transformed and projected? More detail and examples here would greatly expand the utility of this tool.

We have added additional text to the methods, and supplementary files (Table S1), to describe the single-cell data projections. We have also developed a vignette that is accessible from the Stemformatics web page. This vignette describes in more detail recommendations for aggregation of single-cell data, before projection to the atlas. This can be accessed at http://stemformatics.org/static_html/atlas_vignette/Stemformatics_atlas_projection_vignette.html.

I cannot find Galen et al. in the list of datasets. (Also general problem even for datasets listed: I can't find their placement on the plot.)

We've taken on this feedback by modifying the Stemformatics 'find a dataset' feature to allow two ways to highlight a dataset of interest. Firstly, we've added a tier in the 'colour by' pull down menu to include 'colour by dataset'. This lists all of the datasets included in the construction of the atlas and allows for users to recolour any individual dataset as needed using the pencil tool on the legend. The default colours indicate the platform that the experiment was profiled on. Secondly, the original search box is kept, with samples highlighted in black on the atlas. Clicking on any of these samples then allows the users to navigate to the original dataset an explore gene just in that experimental series, which also allows the user to better visualise specific experimental conditions.
For the Galen et al dataset, which was not used in the construction of the atlas, but used to exemplify projection of external data, we've added a table of dataset accessions that distinguishes between samples used for construction, and samples used for projection. Externally projected samples are not saved in the Stemformatics platform. (Table S1).

Professor Christine Wells
Director
Centre for Stem Cell Systems
School of Biomedical Sciences, Faculty of Medicine, Dentistry and Health
30 Royal Parade, The University of Melbourne, Victoria 3010 Australia
T: +61 3 83443795 | M: +61 478306783 | E: wells.c@unimelb.edu.au | W: Stemformatics.org | **unimelb.edu.au**

6. Blood is special, in its level of cell type characterization, ease of sampling, and availability of cell surface markers to sort for particular populations. Can similar methods be used to create similar integrated datasets for other tissues?

We don't have any evidence that blood is special in terms of data complexity, but we agree with the reviewer that it provided a good proof of principle because of the high level of phenotype information available to us. We have tested the method on less curated data, including all of the human samples in the Stemformatics database (currently at 12, 868 samples). Figure 1 is this real-life example, and we do observe partitioning of stromal, pluripotent and blood cell types. As the reviewer implies, the rate-limiting step is review and annotation of the metadata needed to interpret such an atlas. Certainly, we plan to use the method to assess pluripotent cell types and their derivatives, but this will be the focus of a future study. The conditions needed for external users to construct their own atlas using this method are addressed specifically in the discussion, including additional information provided against the reviewers point 3 and point 4.

Minor points

- "iMAC" is probably not a good choice of name for one atlas - I imagine Apple would take issue with this if the tool become popular enough, I would advise changing it
We accept the potential for confusion and have now changed this name to "Myeloid" atlas.

- not clear online how to find a dataset on the plot, e.g. If I click "find dataset" then choose one, it does not show up on the plot?
We have addressed this in detail in response to point 5.

Professor Christine Wells
Director
Centre for Stem Cell Systems
School of Biomedical Sciences, Faculty of Medicine, Dentistry and Health
30 Royal Parade, The University of Melbourne, Victoria 3010 Australia
T: +61 3 83443795 | M: +61 478306783 | E: wells.c@unimelb.edu.au | W: Stemformatics.org | **unimelb.edu.au**
